# Antiproliferative and apoptotic effects of telmisartan in human colon cancer cells

**DOI:** 10.3892/ol.2014.2592

**Published:** 2014-10-09

**Authors:** LUCAS D. LEE, BENJAMIN MAFURA, JOHANNES C. LAUSCHER, HENDRIK SEELIGER, MARTIN E. KREIS, JÖRN GRÖNE

**Affiliations:** Department of General, Visceral and Vascular Surgery, Campus Benjamin Franklin, Charité-University of Medicine Berlin, Berlin D-12203, Germany

**Keywords:** apoptosis, peroxisome proliferator-activated receptor γ, angiotensin I receptor blocker, colon cancer, antiproliferative, telmisartan

## Abstract

Telmisartan is an angiotensin I (AT_1_) receptor blocker used in the treatment of essential hypertension, with partial peroxisome proliferator-activated receptor γ (PPARγ) agonism. In prior studies, PPARγ activation led to apoptosis and cell cycle inhibition in various cancer cells. The aim of the present study was to investigate the potential antiproliferative and apoptotic effects of telmisartan by partially activating PPARγ. HT-29, SW-480 and SW-620 cells were incubated with telmisartan (0.2–5 μM) or the full agonist, pioglitazone (0.2–5.0 μM). The antiproliferative and apoptotic effects of telmisartan in the human colon cancer cells were significant at therapeutic serum concentrations, and telmisartan exhibited a potency at least equivalent to the full PPARγ agonist, pioglitazone. The antiproliferative and apoptotic effects of pioglitazone in the human colon cancer cells were not completely deregulated by PPARγ blockade with GW9662. In the telmisartan-treated cells, PPARγ blockade resulted in an increased antiproliferative and apoptotic effect. These effects are not entirely explained by PPARγ activation, however, possible hypotheses that require further experimental investigation are as follows: i) Ligand-independent PPARγ activation through the activation-function 1 domain; ii) a PPARγ-independent mechanism; or iii) independent antiproliferative and apoptotic effects through GW9662.

## Introduction

Telmisartan is an antihypertensive drug that exerts its effect by antagonizing the angiotensin I (AT_1_) receptor blocker (ARB). Beyond this mechanism, Shupp *et al* (1) revealed partial peroxisome proliferator-activated receptor (PPAR) agonism in 3T3-L1 preadipocytes. PPAR belongs to the steroid hormone receptor superfamily (2) and can be divided into three types: PPARα, PPARβ/δ and PPARγ. Alternative splicing of PPARγ leads to four isoforms: PPARγ_1–4_. Translating the mRNA of PPARγ_1,3,4_ results in an identical protein (3). By contrast, the PPARγ_2_ protein differs from PPARγ_1_ due to the additional 30 amino acids at its N-terminal, and it predominantly exists in adipocytes, but is also highly expressed in colon epithelium, pancreatic β cells, endothelial tissue and macrophages. In addition, PPARγ_1_ is present in human urological cancer cells (i.e., renal cell, prostate, bladder and testicular cancer) (4) and colon cancer cells (5), where ligand-dependent activation by antidiabetic drugs, such as thiazolidinediones, which include pioglitazone, rosiglitazone, troglitazone and ciglitazone, leads to apoptosis (6) and an antiproliferative effect (7).

Arterial hypertension and colorectal cancer (CRC) have a high prevalence in industrialized nations, with an estimated proportion of 25% for hypertension and an age standardized incidence (Europe) for CRC of 67.0% for males and 44.5% for females in Germany (8). Synchronous manifestation is common, however, at present, it is unclear whether telmisartan partially activates PPARγ_1_ in human colon cancer cells and whether its partial agonism is sufficient for inhibiting proliferation and stimulating apoptosis in a significant manner.

## Materials and methods

### Cell culture

Human colon cancer cells (HT-29, SW-480 and SW-620) were obtained from the American Type Culture Collection (Rockville, MD, USA). The HT-29 and SW-480 cells originated from well-differentiated colorectal adenocarcinoma (9) and the SW-620 cells were derived from colorectal lymph node metastasis. The cell lines were maintained in RPMI-1640 culture medium (Life Technologies, Darmstadt, Germany), containing 10% fetal calf serum and 1% penicillin and streptomycin (all Biochrom AG Biotechnologie, Berlin, Germany), at 37°C in a 5% CO_2_ humidified atmosphere. Each cell line was treated with telmisartan (0.2–5.0 μM; Boehringer Ingelheim, Ingelheim, Germany) or the full PPARγ agonist, pioglitazone (0.2–5.0 μM; Zhejiang Huahai Pharmaceutical Co., Ltd., Zhejiang, China), as a positive control for 24 h. Pioglitazone and telmisartan were each dissolved in 0.05% dimethyl sulfoxide (DMSO; Carl Roth GmbH & Co., KG, Karlsruhe, Germany). DMSO served as a negative control.

### MTT cytotoxicity assay

The measured activity of mitochondrial succinate dehydrogenase quantitatively determines the degree of cytotoxicity. The enzyme converts tetrazolium salt (MTT; Sigma-Aldrich Chemie GmbH, Taufkirchen, Germany) into a blue dye, and the absorption is measured. Higher levels of absorption indicate higher levels of enzyme activity and increased cell viability. According to the manufacturer’s instructions, the cells were detached using 3 ml trypsin and then transferred to a 96-well microplate. Each well contained 3×10^3^ cells per 0.2 ml, and the cells were incubated at 37°C and 95% relative atmospheric humidity for 24 h. Following three days of incubation with 100 μl medium and 10 μl MTT solution [50 mg/10 ml phosphate-buffered saline (PBS); PAA Laboratories GmbH, Cölbe, Germany ], the cells were incubated for a further four hours. Cell lysis was initiated by the addition of 100 μl SDS (10%; 5 g/50 ml double-distilled water) purchased from Carl Roth GmbH & Co., KG. The cell suspension was incubated overnight and the absorbance at 570 nm was measured on the following day with a reference at 650 nm.

### Cell count

Following 24 h of incubation with DMSO, pioglitazone and telmisartan in the presence or absence of the PPARγ antagonist, GW9662, the cell culture medium was carefully removed, followed by multiple PBS-washing cycles. Cell counting was performed using a Neubauer cell count chamber (Brand GmbH + Co., KG, Wertheim, Germany).

### Caspase 3–7 assay

The Caspase-Glo^®^ 3/7 assay (AnaSpec Inc., Seraing, Belgium) is a homogeneous, luminescent assay that measures caspase-3 and -7 activity. The assay provides a luminogenic caspase-3/7 substrate, which contains the Asp-Glu-Val-Asp tetrapeptide sequence, in a reagent optimized for caspase activity, luciferase activity and cell lysis. Adding a single Caspase-Glo^®^ 3/7 reagent in an ‘add-mix-measure’ format results in cell lysis, followed by caspase cleavage of the substrate and generation of a ‘glow-type’ luminescent signal, produced by luciferase. Luminescence is proportional to the amount of caspase activity present.

### Quantitative polymerase chain reacton (qPCR)

Primers were purchased from TIB Molbiol Syntheselabor GmbH (Berlin, Germany) with the following sequences: PPARγ Sense, 5′-CAAGCCCTTCACTACTGTTG-3′ and antisense, 5′-CTTTATCTCCACAGACACG-3′; and AT_1_ receptor sense, 5′-ACAGCTTGGTGGTGATAGTC-3′ and antisense, 5′-CAATGCTGAGACACGTGAG-3′. The qPCR was performed using the Roche LightCycler^®^ carousel-based system (Roche Diagnostics GmbH, Mannheim, Germany) under the following conditions: Activation at 95°C for 10 min; 40 cycles of amplification at 95°C for 5 sec, 64°C for 10 sec and 72°C for 20 sec; and a brief melting curve analysis at 95°C and 65°C for 15 sec, followed by an increase of 0.1°C/sec to 95°C.

### Statistical analyses

Statistical significance was determined using the independent Student t-test, SPSS Version 19.0.1 (IBM, Armonk, NY, USA). P<0.05 was considered to indicate a statistically significant difference.

## Results

### Telmisartan affects cell viability of human colon cancer cells

**Figure 1:**
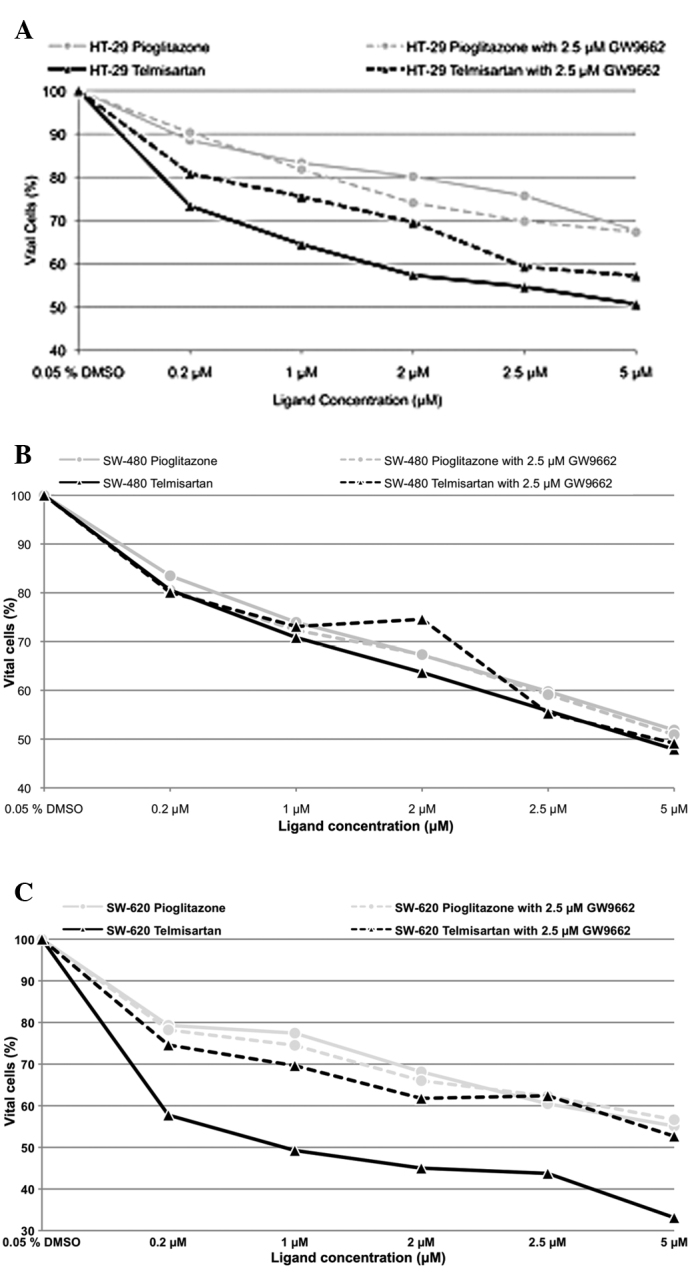
Determination of antiproliferative effect by cell count in a Neubauer cell chamber. (A) HT-29, (B) SW-480 and (C) SW-620 cells were incubated with pioglitazone (0–5 μM) and telmisartan (0–5 μM) for 24 h. The percentage of vital cells revealed that the antiproliferative effect of the partial PPARγ-agonist, telmisartan, was greater than that of the full PPARγ agonist, pioglitazone. PPARγ, peroxizome proliferator-activated receptor γ; DMSO, dimethyl sulfoxide.

The incubation of the human colon cancer cell lines with pioglitazone and telmisartan exhibited a dose-dependent effect on cell viability (Fig. 1), while incubation of the HT-29 cells with 0.2 μM telmisartan resulted in 98.65% cell viability (P>0.05). Additional blocking of PPARγ with 2.5 μM GW9662 resulted in 96.60% (P>0.05) cell viability (telmisartan vs. telmisartan combined with GW9662; P>0.05). In the SW-480 and SW-620 cells, incubation with 0.2 μM telmisartan alone resulted in a cell viability of 98.13 and 96.33% (P>0.05), respectively. In the presence of 2.5 μM GW9662, the cell viability was reduced to 88.53 and 86.77% (P<0.001). A concentration of 0.2 μM pioglitazone alone resulted in 97.31, 99.63 and 96.74% cell viability for the HT-29, SW-480 and SW-620 cells, respectively (P>0.05). Additional blocking of PPAR with 2.5 μM GW9662 resulted in 98.10 (P>0.05), 95.23 and 91.70% (P<0.001) cell viability for the HT-29, SW-480 and SW-620 cells, respectively. No significant differences were identified with regard to the effect on cell viability between telmisartan and pioglitazone regardless of the use of GW9662 in all three human colon cell lines.

### Antiproliferative effect of telmisartan in human colon cancer cells

**Figure 2:**
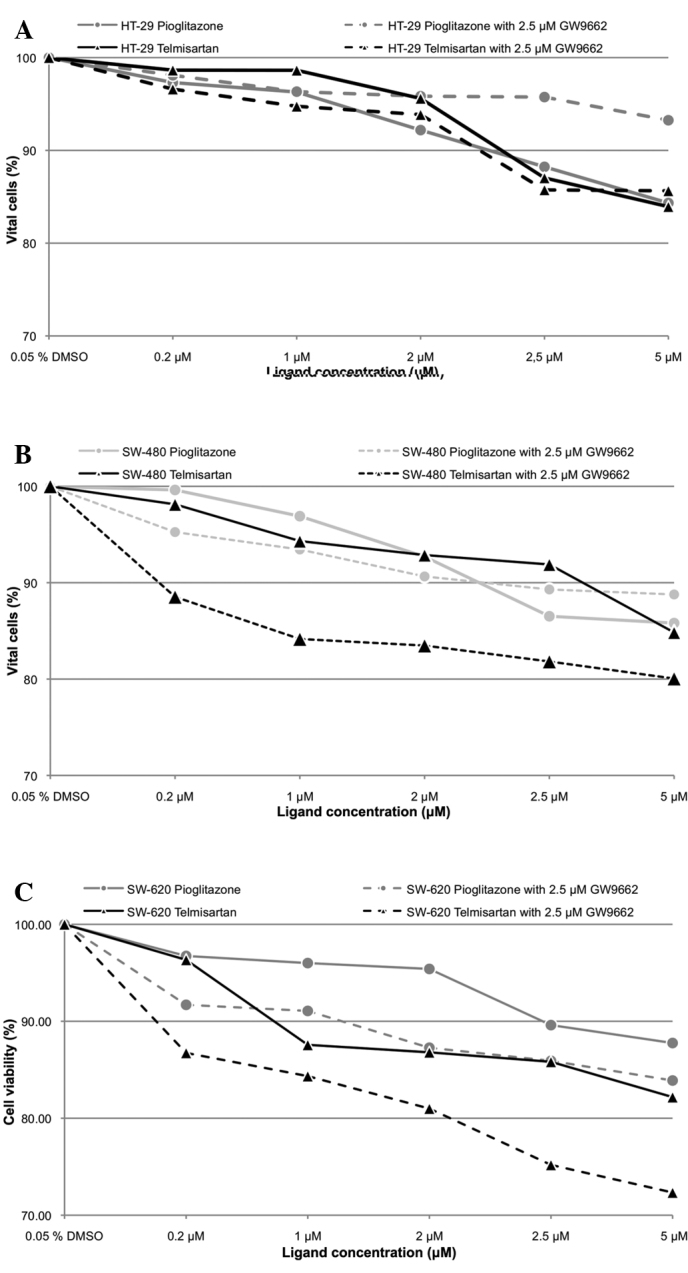
Determination of cell viability by MTT assay. (A) HT29, (B) SW-480 and (C) SW620 cells were incubated with pioglitazone (0.2–5 μM) and telmisartan (0.2–5 μM) for 24 h. The percentages of vital cells are shown with respect to ligand concentration. DMSO, dimethyl sulfoxide; Pio, pioglitazone.

Telmisartan and pioglitazone inhibited cell proliferation in a dose-dependent manner (Fig. 2). Telmisartan (0.2 μM) significantly reduced cell survival in the HT-29, SW-480 and SW-620 cells (73.33, 80.56 and 57.75%, respectively; P<0.001). Additional PPARγ blockage with 2.5 μM GW9662 led to a significant increase in cell survival in the HT-29 and SW-620 cells (80.78 and 74.59%, respectively; P<0.001), however, this increase was not observed in the SW-480 cells (80.08%; P>0.05). The incubation of the HT-29, SW-480 and SW-620 cells with 0.2 μM pioglitazone resulted in a significantly reduced cell count (88.51, 83.49 and 79.30%; P<0.001). However, no significant differences in cell count were observed by adding 2.5 μM GW9662 (90.27% for HT-29, 80.56% for SW-480 and 78.21% for SW-620; P>0.05). The difference in the antiproliferative effect between pioglitazone and telmisartan was not significant in the HT-29 cells, regardless of the use of GW9662. By contrast, the difference in the antiproliferative effect was significant in the SW-480 cells (P<0.001; P<0.05 with GW9662), as well as in the SW-620 cells in the absence of GW9662 (P<0.05; P>0.05 with GW9662).

### Telmisartan induces apoptosis

**Figure 3:**
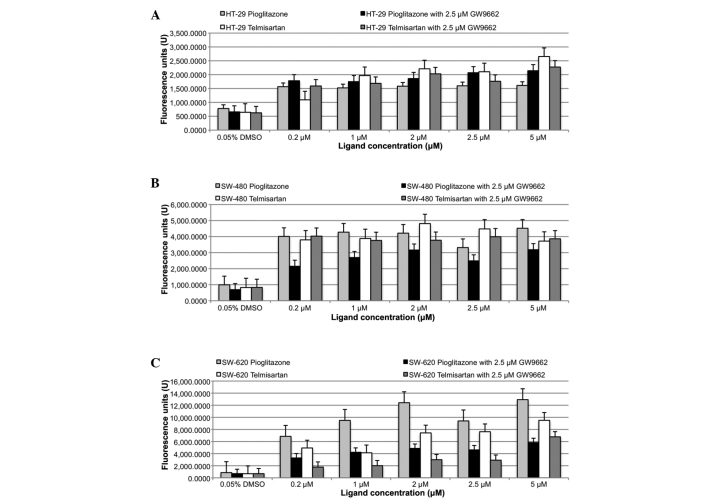
Caspase 3/7 assay of (A) HT-29, (B) SW-480 and (C) SW-620 cells following 24 h of incubation with pioglitazone and telmisartan, in the absence or presence of 2.5 μM of the peroxizome proliferator-activated receptor γ antagonist, GW9662. DMSO, dimethyl sulfoxide.

The induction of apoptosis was determined by the caspase 3/7 assay (Fig. 3). Significant caspase 3/7 activation was measured with 0.2 μM pioglitazone and telmisartan (P<0.05) in all three colon cancer cell lines (HT-29, SW-480 and SW-620). However, no significant difference was observed between the apoptotic effects of telmisartan and pioglitazone. Similarly, the addition of 2.5 μM of the PPARγ blocker, GW9662, also resulted in significant caspase 3/7 activation (P<0.05) in all three colon cancer cell lines. No significant differences were identified in caspase 3/7 activation between telmisartan alone versus telmisartan in combination with GW9662, and the same was true for pioglitazone.

### Telmisartan downregulates PPARγ and upregulates cystatin A (CSTA)

**Figure 4:**
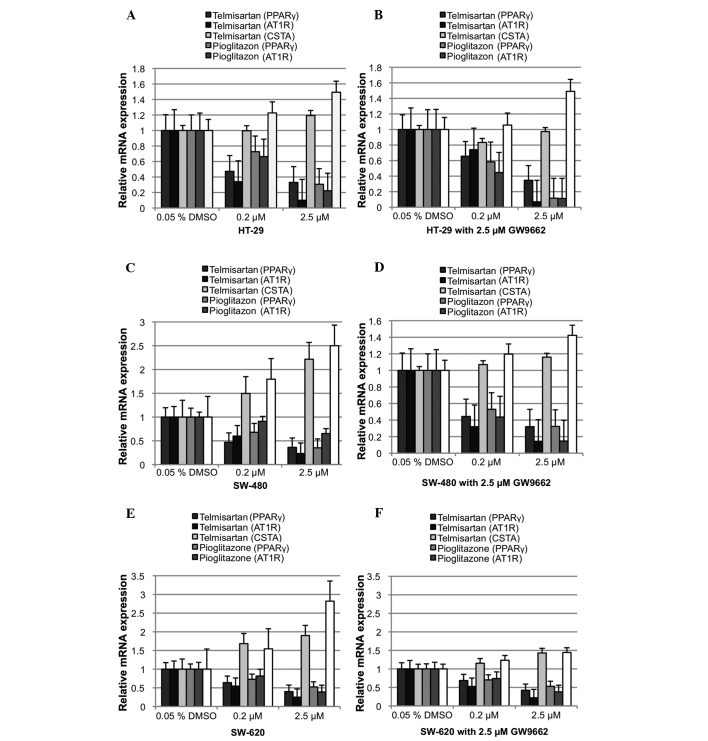
Relative mRNA expression of PPARγ, the PPARγ target gene, CSTA and AT1R following 24 h of incubation with DMSO, pioglitazone and telmisartan. (A) HT-29 without and (B) with the PPARγ antagonist, GW9662. (C) SW-480 without and (D) with the PPARγ antagonist, GW9662. (E) SW-620 without and (F) with the PPARγ antagonist, GW9662. PPARγ, peroxizome proliferator-activated receptor γ; DMSO, dimethyl sulfoxide; CSTA, cystatin A; AT1R, angitensin I receptor.

PPARγ was found to downregulate its relative mRNA expression following ligand activation (Fig. 4). Relative PPARγ mRNA expression was significantly downregulated in the HT-29, SW-480 and SW-620 cells with 0.2 μM telmisartan (Δ0.53, Δ0.53 and Δ0.36; P<0.05). The addition of 2.5 μM GW9662 also resulted in a significant downregulation of relative PPARγ mRNA expression (HT-29, Δ0.34; SW-480, Δ0.67; and SW-620, Δ0.31; P<0.05). However, no significant differences were observed between telmisartan alone and telmisartan combined with GW9662. The incubation of the HT-29, SW-480 and SW-620 cells with 0.2 μM pioglitazone also resulted in a significant downregulation of relative PPARγ mRNA expression (Δ0.27, Δ0.32 and Δ0.27, respectively; P<0.05). PPARγ blockage did not neutralize the effect of pioglitazone on PPARγ mRNA expression in all three colon cancer cell lines (HT-29, Δ0.42; SW-480, Δ0.48; and SW-620, 0.27; P<0.05). AT_1_ receptor mRNA was downregulated significantly through telmisartan in all three colon cancer cell lines (P<0.05), as predicted. Notably, pioglitazone had the same effect in all three colon cancer cell lines (P<0.05). CSTA, a well-known PPARγ_1_ target gene, which is upregulated upon PPARγ activation, was used as a positive control. Relative CSTA mRNA expression was not significantly affected by 0.2 μM telmisartan or telmisartan combined with 2.5 μM GW9662 in the HT-29 and SW-620 cells. While significant upregulation was observed in the SW-480 cells (Δ0.5; P<0.05), the addition of 2.5 μM GW9662 (P>0.05) did not cause this result. Relative CSTA mRNA expression in the HT-29 cells was not significantly affected by 0.2 μM pioglitazone, alone or in combination with GW9662. By contrast, relative CSTA mRNA expression in the SW-620 cells was significantly upregulated (Δ0.50 alone and Δ0.20 with GW9662; P<0.05).

## Discussion

The thiazolidinedione, pioglitazone, is an oral antidiabetic drug that enhances insulin sensitivity through PPARγ activation. However, it also activates PPARγ in colon cancer cells *in vitro*, inducing the suppression, differentiation, apoptosis and reversal of malignant changes (7,10). Telmisartan is an ARB and a common antihypertensive drug with partial PPARγ-agonism (1). Therefore, it is of note whether telmisartan partially activates PPARγ_1_ in colon cancer cells, subsequently inducing a reduction in cell viability, inhibiting cell proliferation and inducing apoptosis.

Following the oral intake of 20–120 mg telmisartan by hypertensive patients, the therapeutic serum concentration has been measured to range between 0.035 and 1.036 mM (11). Therefore, in the present study, various colon cancer cells were incubated with telmisartan and pioglitazone in comparable concentrations ranging between 0.2 and 5.0 μM. This resulted in a dose-dependent reduction in cell viability, the inhibition of cell proliferation and the induction of apoptosis in all three colon cancer cell lines. As predicted, the effect of the full agonist, pioglitazone, was diminished by adding the synthetic PPARγ antagonist, GW9662. PPARγ blockage with GW9662 in the presence of telmisartan as a partial PPARγ agonist did not lead to the diminished inhibition of the cell proliferation and cell viability of pioglitazone. However, the reduction in cell viability and the antiproliferative effect in the SW-480 and SW-620 cells were further increased compared with use of telmisartan alone. Furthermore, telmisartan exhibited an apoptotic effect equivalent to the full PPARγ agonist, pioglitazone. Contrary to the prediction of a decreased apoptotic effect following the addition of the PPARγ GW9662, a significant induction of caspase 3/7 was observed, indicating PPARγ-independent apoptosis induction. Following ligand-dependent activation, the PPARγ mRNA level was characteristically downregulated as a negative feedback mechanism, whilst upregulating the mRNA of its target genes, including CSTA. Incubation with pioglitazone or telmisartan only significantly downregulated relative PPARγ mRNA expression in all three colon cancer cell lines, while upregulating CSTA in an evidently dose-dependent manner. However, the additional blockage with 2.5 μM GW9662 resulted in a significant downregulation in relative PPARγ mRNA expression.

In conclusion, the present study showed that telmisartan reduced cell viability and inhibited cell proliferation in selected colon cancer cell lines in a dose-dependent manner. Furthermore, telmisartan showed a greater effect than pioglitazone in the SW-480 and SW-620 cells, particularly in the presence of the PPARγ blocker, GW9662. The apoptotic effect of telmisartan was close to the full PPARγ agonist, pioglitazone, in all cell lines and was not inhibited by GW9662. PPARγ and CSTA mRNA expression appeared to be affected by the presence of GW9662. Therefore, the observed reduction in cell viability, the inhibition of cell proliferation and the induction of apoptosis by telmisartan cannot be completely explained by ligand-dependent PPARγ_1_ activation.

Telmisartan may be an alternative drug for patients presenting with arterial hypertension and colon cancer in their medical history. Understanding its mechanism of action may lead to similar pleiotropic drugs with increased potency.

## References

[1] Schupp M, Janke J, Clasen R, Unger T, Kintscher U (2004). Angiotensin type 1 receptor blockers induce peroxisome proliferator-activated receptor-gamma activity. Circulation.

[2] Mangelsdorf DJ, Thummel C, Beato M (1995). The nuclear receptor superfamily: the second decade. Cell.

[3] Fajas L, Fruchart JC, Auwerx J (1998). PPARgamma3 mRNA: a distinct PPARgamma mRNA subtype transcribed from an independent promoter. FEBS Lett.

[4] Matsuyama M, Funao K, Kuratsukuri K (2010). Telmisartan inhibits human urological cancer cell growth through early apoptosis. Exp Ther Med.

[5] Fajas L, Auboeuf D, Raspé E (1997). The organization, promoter analysis, and expression of the human PPARgamma gene. J Biol Chem.

[6] Bull AW (2003). The role of peroxisome proliferator-activated receptor gamma in colon cancer and inflammatory bowel disease. Arch Pathol Lab Med.

[7] Sarraf P, Mueller E, Jones D (1998). Differentiation and reversal of malignant changes in colon cancer through PPARgamma. Nat Med.

[8] Bertz J, Dahm S, Haberland J, Kraywinkel K, Kurth B-M, Wolf U (2010). Spread of Cancer in Germany. Development in the prevalence between 1990 and 2010.

[9] Semple TU, Quinn LA, Woods LK, Moore GE (1978). Tumor and lymphoid cell lines from a patient with carcinoma of the colon for a cytotoxicity model. Cancer Res.

[10] Kato M, Kusumi T, Tsuchida S, Tanaka M, Sasaki M, Kudo H (2004). Induction of differentiation and peroxisome proliferator-activated receptor gamma expression in colon cancer cell lines by troglitazone. J Cancer Res Clin Oncol.

[11] Stangier J, Su CA, Roth W (2000). Pharmacokinetics of orally and intravenously administered telmisartan in healthy young and elderly volunteers and in hypertensive patients. J Int Med Res.

